# Supplementary material for: Long-term brain pressure monitoring via a discrete microimplant; a first-in-human safety and initial efficacy trial in adults and children with hydrocephalus
Source: Nat Commun. 2026 Apr 2;17:3158. doi: 10.1038/s41467-026-70864-8 (PMC13046746; doi:10.1038/s41467-026-70864-8)
Supplement: Supplementary file 1 — Reporting Summary [file 41467_2026_70864_MOESM1_ESM.pdf]

nature portfolio

Corresponding author(s): Malpas SC

Last updated by author(s): Feb 10, 2026

Reporting Summary

Nature Portfolio wishes to improve the reproducibility of the work that we publish. This form provides structure for consistency and transparency in reporting. For further information on Nature Portfolio policies, see our Editorial Policies and the Editorial Policy Checklist.

Statistics

For all statistical analyses, confirm that the following items are present in the figure legend, table legend, main text, or Methods section.

n/a	Confirmed	
☐	☒	The exact sample size (*n*) for each experimental group/condition, given as a discrete number and unit of measurement
☐	☒	A statement on whether measurements were taken from distinct samples or whether the same sample was measured repeatedly
☒	☐	The statistical test(s) used AND whether they are one- or two-sided<br>*Only common tests should be described solely by name; describe more complex techniques in the Methods section.*
☒	☐	A description of all covariates tested
☒	☐	A description of any assumptions or corrections, such as tests of normality and adjustment for multiple comparisons
☒	☐	A full description of the statistical parameters including central tendency (e.g. means) or other basic estimates (e.g. regression coefficient) AND variation (e.g. standard deviation) or associated estimates of uncertainty (e.g. confidence intervals)
☒	☐	For null hypothesis testing, the test statistic (e.g. *F*, *t*, *r*) with confidence intervals, effect sizes, degrees of freedom and *P* value noted<br>*Give P values as exact values whenever suitable.*
☒	☐	For Bayesian analysis, information on the choice of priors and Markov chain Monte Carlo settings
☒	☐	For hierarchical and complex designs, identification of the appropriate level for tests and full reporting of outcomes
☒	☐	Estimates of effect sizes (e.g. Cohen's *d*, Pearson's *r*), indicating how they were calculated

Our web collection on statistics for biologists contains articles on many of the points above.

Software and code

Policy information about availability of computer code

Data collection	No specialised software was used
Data analysis	No specialised software was used

For manuscripts utilizing custom algorithms or software that are central to the research but not yet described in published literature, software must be made available to editors and reviewers. We strongly encourage code deposition in a community repository (e.g. GitHub). See the Nature Portfolio guidelines for submitting code & software for further information.

Data

Policy information about availability of data

All manuscripts must include a data availability statement. This statement should provide the following information, where applicable:

- Accession codes, unique identifiers, or web links for publicly available datasets
- A description of any restrictions on data availability
- For clinical datasets or third party data, please ensure that the statement adheres to our policy

The anonymised data collected is available as open data via the University of Auckland online data repository (links included in manuscript)

Research involving human participants, their data, or biological material

Policy information about studies with human participants or human data. See also policy information about sex, gender (identity/presentation), and sexual orientation and race, ethnicity and racism.

Reporting on sex and gender	Gender is reported in the manuscript
Reporting on race, ethnicity, or other socially relevant groupings	There is no report on race, ethnicity etc as the study involves only a small number of subjects and such analysis is inappropriate given the same size
Population characteristics	The age range of subjects is reported
Recruitment	Subjects were recruited prior to their surgery. Subjects gave written informed consent
Ethics oversight	The protocol was approved by the Health and Disability Ethics committee of NZ and given locality approval by the Auckland City hospital. During the study the data was reviewed by an independent data safety monitoring committee. The operation of the trial conformed to Good Clinical Practice guidelines

Note that full information on the approval of the study protocol must also be provided in the manuscript.

Field-specific reporting

Please select the one below that is the best fit for your research. If you are not sure, read the appropriate sections before making your selection.

☒ Life sciences ☐ Behavioural & social sciences ☐ Ecological, evolutionary & environmental sciences

For a reference copy of the document with all sections, see nature.com/documents/nr-reporting-summary-flat.pdf

Life sciences study design

All studies must disclose on these points even when the disclosure is negative.

Sample size	The clinical aspect to this study is primarily a safety and initial efficacy. The sample size was justified based on the requirements to show a lack of adverse events attributable to the device
Data exclusions	No data was excluded
Replication	NA
Randomization	The study design was a single arm open study ie not randomised
Blinding	The study design was a single arm open study and this blinding was not relevant to our study

Reporting for specific materials, systems and methods

We require information from authors about some types of materials, experimental systems and methods used in many studies. Here, indicate whether each material, system or method listed is relevant to your study. If you are not sure if a list item applies to your research, read the appropriate section before selecting a response.

Materials & experimental systems

n/a	Involved in the study	
☒	☐	Antibodies
☒	☐	Eukaryotic cell lines
☒	☐	Palaeontology and archaeology
☒	☐	Animals and other organisms
☐	☒	Clinical data
☒	☐	Dual use research of concern
☒	☐	Plants

Methods

n/a	Involved in the study	
☒	☐	ChIP-seq
☒	☐	Flow cytometry
☒	☐	MRI-based neuroimaging

Clinical data

Policy information about clinical studies

All manuscripts should comply with the ICMJE guidelines for publication of clinical research and a completed CONSORT checklist must be included with all submissions.

Clinical trial registration

NCT06402786

Study protocol

First-in-human Trial of Home Brain Pressure Measured Using Kitea ICP Sensor, Placed During Hydrocephalus Shunt Surgery. (HomeICP)

Data collection

Auckland City Hospital,Inclusion Criteria:

Participants with a condition requiring cerebral spinal fluid shunting who are undergoing initial shunt placement or shunt revision surgery.
Adults: Age > 16 years; Children: Age >1 and <15,
Exclusion Criteria:

Adults: Unable to give informed consent
Paediatric: Failure to obtain assent in a cognitively competent child along with parental consent or parent unable to consent.
Cortical mantle < 20 mm
Neurologic or other condition that would prevent compliance with protocol
Terminal illness with expected survival < 1 year
Unable to participate in follow-up for 3 months (e.g., travelling overseas for an extended period)
Underlying medical condition that would make the participant more prone to surgical infections
Other active implanted medical devices e.g. pacemaker, cochlear implant, implantable cardioverter defibrillator, deep brain stimulation devices
Participants with conditions likely to require radiation therapy

Outcomes

The primary end point for this study is the proportion of recruited participants who have no safety or tolerability issues in the 3 months post-surgery as assessed by a lack of serious adverse events attributable to the Kitea ICP System or participants requesting device removal.Wireless home ICP monitoring will be considered successful if 90% of the attempted ICP measures are able to be obtained.As part of the protocol participants will be asked to make daily ICP measurements using the Kieta ICP system for the first 2 weeks after surgery and then make measurements at least every second day for the remainder of the 3 months. A participant survey at 3 months will collect participant feedback on the perceived burden of making those measurements.

Plants

Seed stocks

Report on the source of all seed stocks or other plant material used. If applicable, state the seed stock centre and catalogue number. If plant specimens were collected from the field, describe the collection location, date and sampling procedures.

Novel plant genotypes

Describe the methods by which all novel plant genotypes were produced. This includes those generated by transgenic approaches, gene editing, chemical/radiation-based mutagenesis and hybridization. For transgenic lines, describe the transformation method, the number of independent lines analyzed and the generation upon which experiments were performed. For gene-edited lines, describe the editor used, the endogenous sequence targeted for editing, the targeting guide RNA sequence (if applicable) and how the editor was applied.

Authentication

Describe any authentication procedures for each seed stock used or novel genotype generated. Describe any experiments used to assess the effect of a mutation and, where applicable, how potential secondary effects (e.g. second site T-DNA insertions, mosiacism, off-target gene editing) were examined.
